# Surgical Technique: Endoscopic Endonasal Transphenoidal Resection of a Large Suprasellar Mixed Germ Cell Tumor

**DOI:** 10.7759/cureus.503

**Published:** 2016-02-21

**Authors:** Olaide Ajayi, Vikram Chakravarthy, George Hanna, Kennethy DeLos Reyes

**Affiliations:** 1 Department of Neurosurgery, Loma Linda University Medical Center

**Keywords:** endoscopy, craniopharyngioma

## Abstract

The endoscopic endonasal transphenoidal approach has proven to be a very versatile surgical approach for the resection of small midline skull base tumors. This is due to its minimally invasive nature, the potentially fewer neurological complications, and lower morbidity in comparison to traditional craniotomies. This surgical approach has been less commonly utilized for large midline tumors such as suprasellar germ cell tumors, due to numerous reasons including the surgeon’s comfort with the surgical approach, a higher chance of postoperative cerebrospinal fluid (CSF) leak, limited visualization due to arterial/venous bleeding, and limited working space. We present our surgical technique in the case of a large suprasellar and third ventricular mixed germ cell tumor that was resected via an endoscopic endonasal approach with favorable neurological outcome and no postoperative CSF leak.

## Introduction

Intracranial germ cell tumors (GCTs) are rare neoplasms usually localized in the pineal and suprasellar regions and comprise approximately 1% of all primary brain tumors in adults [1]. They are more common in children, making up 15% of childhood intracranial tumors in Japan and 7% in the West [2]. The peak incidence of germ cell tumors is in the second decade with a male to female ratio of 2.5:1 [1].

For sellar and suprasellar germ cell tumors that are best treated with surgical resection, pterional, orbitozygomatic, and bifrontal craniotomies have traditionally been the most utilized surgical approaches. However, the versatility of the endoscopic transnasal transphenoidal approach provides a less invasive option for the surgical resection of germ cell tumors located in the sellar, suprasellar, and parasellar regions. Presented here is a case report of a large suprasellar and third ventricular mixed germ cell tumor that was resected via an endonasal endoscopic transphenoidal approach, with a favorable surgical outcome, highlighting the safety and utility of this approach to the skull base. Informed patient consent was obtained for this study. Based on our review of current literature, there are only two other reported cases in which an endonasal endoscopic transphenoidal approach was employed for the resection of a suprasellar germ cell tumor. 

## Case presentation

**Figure 1:**
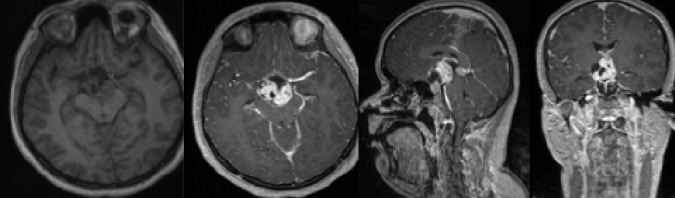
Preoperative Brain MRI

A previously healthy 18-year-old female presented with a three-week history of bitemporal hemianopsia and a one-year history of amenorrhea. Her neurological examination and medical history was otherwise not contributory. The presenting brain magnetic resonance imaging (MRI) is shown below in Figure 1. 

Abnormal laboratory values included an elevated prolactin level and decreased free T4, B-HCG, FSH, ACTH, LH, and AM cortisol.

### Surgical approach

A combined neurosurgery and otolaryngology approach with neuronavigation was utilized. A lumbar drain was placed for CSF diversion. Using a rigid 0-degree endoscope for visualization, a nasoseptal flap was harvested. Bilateral posterior ethmoidectomies and septostomy were performed, followed by a wide opening of the sphenoid sinus from the clivus to planum sphenoidale. The bony septum was flattened with a high-speed drill and the medial opticocarotid recesses were identified bilaterally. A craniectomy was performed encompassing the floor of the sella and the anterior portion of the planum sphenoidale, just posterior to the cribriform plate. The sella and planum dura were opened, as was the medial opticocarotid recesses bilaterally to gain lateral access to the suprasellar tumor. Horizontal incisions were made in the dura above and below the superior intracavernous sinus. The dura was reflected in a cruciate fashion and the pituitary gland was noted adjacent to the optic chiasm due to its superior displacement by the tumor. The optic chiasm was then identified superiorly; the tumor and its capsule were resected sequentially and sharply dissected off the optic chiasm, using rigid 0-degree and 30-degree endoscopes for visualization. The closure/skullbase reconstruction was performed using non-suturable dural allograft inlay, a nasoseptal flap, and a dural sealant (DuraSeal® Covidien, Dublin, Ireland).

**Figure 2:**
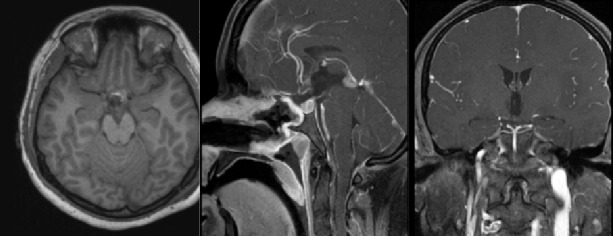
Postoperative Brain MRI

The postoperative brain MRI is shown below in Figure 2. 

### Postoperative course

Intermittent lumbar drainage was utilized for four days and the lumbar drain was discontinued after a clamp trial. A spinal imaging obtained did not show any drop metastases. The patient developed hypopituitarism and diabetes insipidus postoperatively, which were medically treated.

### Pathology

**Figure 3:**
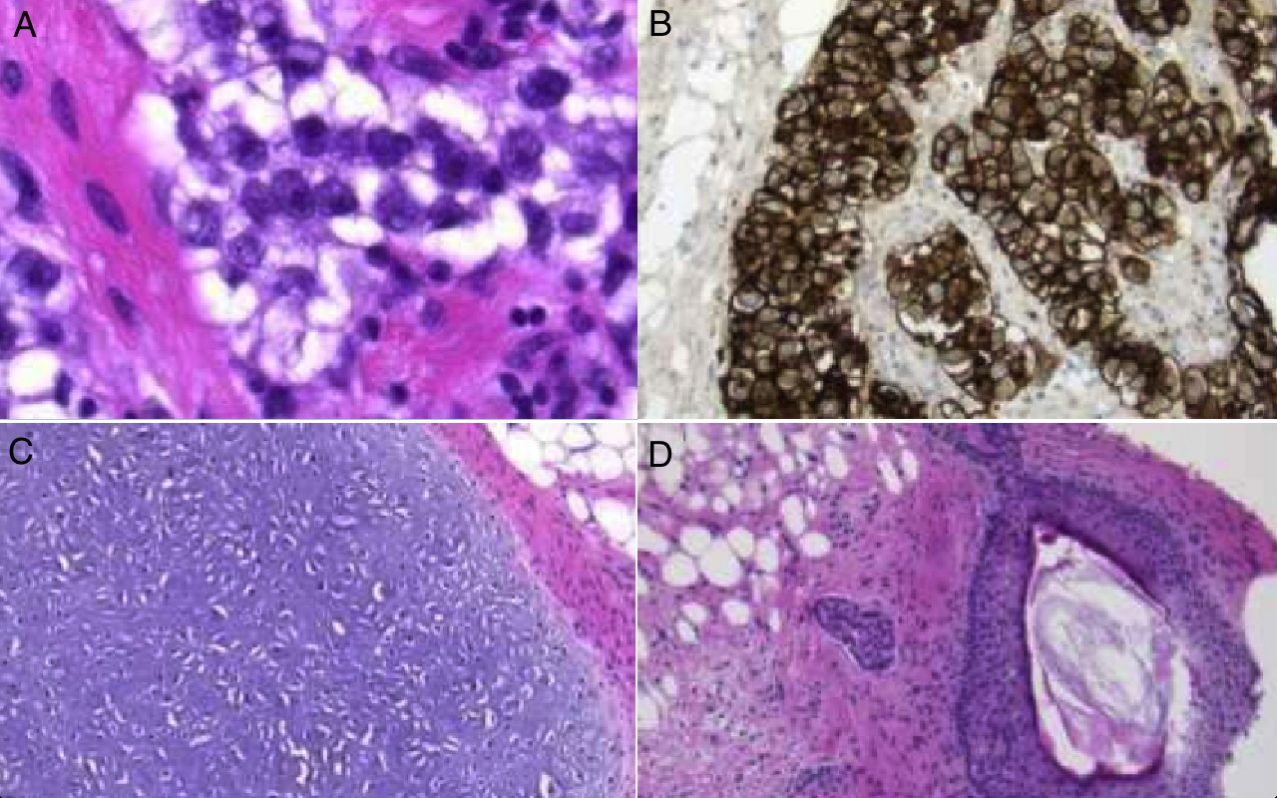
Pathology 3a: Primitive germinoma component 3b: Germinoma component strongly expresses PLAP 3c: Cartilage and adipose tissue 3d: Mature squamous epithelium

The pathology slides highlighted a primitive germinoma component with strong expression of placental alkaline phosphatase (PLAP) and the presence of cartilage, adipose tissue, and mature squamous epithelium in the tumor as shown below in Figure 3. These findings yielded a diagnosis of a mixed germ cell tumor. 

### Follow-up

**Figure 4:**
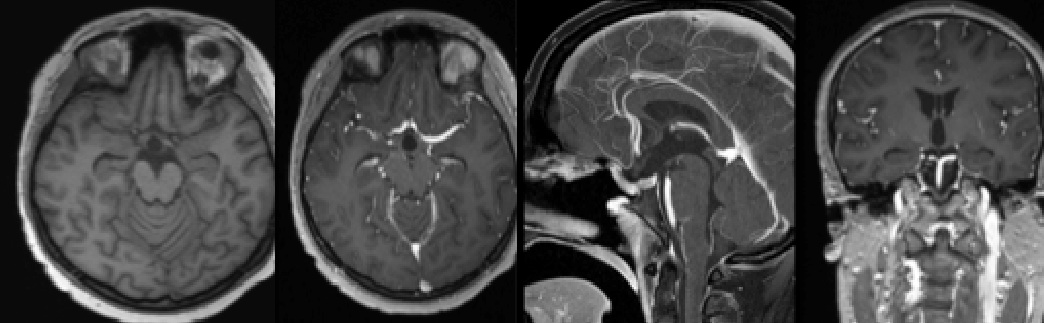
Six-month Postoperative Brain MRI

The patient received induction chemotherapy (with carboplatin and etoposide) and proton therapy with 3420 cGy fractionated over 18 sessions. She reported subjective improvement of her vision at her three-month postoperative visit and still had hypopituitarism requiring hormone replacement by her six-month postoperative visit. A six-month postoperative MRI showed no spinal drop metastases and no definite residual or recurrence of the suprasellar and pineal region masses. Based on formal visual field testing, there was significant improvement of her vision by her six-month postoperative visit. Her six-month postoperative brain MRI is shown below in Figure 4.

## Discussion

Mixed intracranial GCTs are not rare, representing 25–32% of intracranial GCTs [2-3]. According to the World Health Organization classification and the germ cell theory, intracranial GCTs are divided into five histological subtypes of increasingly malignant behavior: germinomas, teratomas, embryonal carcinomas, yolk sac tumors, and choriocarcinomas [2, 4-5]. The treatment recommendations for intracranial germ cell tumors vary from surgical resection to radiotherapy and chemotherapy, depending on the subtype involved. For example, germinomas are treated with radiotherapy, mature teratomas are treated with gross total resection, immature teratomas are treated with gross total resection with adjuvant chemotherapy and radiation therapy, while choriocarcinomas, embryonal carcinomas, and yolk sac tumors are treated with chemotherapy and radiation therapy with surgical resection of residual tumor. In cases of mixed germ cell tumor, the treatment of choice is dependent on the malignant component [1].

Over the past decade it has become clear that the entire ventral skullbase is accessible using an endonasal approach. This has been termed the expanded endonasal approach (EEA) and it provides access to the anterior, middle, and posterior cranial fossa [6]. The EEA can provide the most direct approach to midline tumors by avoiding frontal lobe retraction with its associated temporary (and occasionally permanent) neurological deficits [7], and also limiting manipulation of supra- and parasellar structures such as the optic chiasm and carotid arteries. Angled endoscopes can provide access to areas that would have been impossible to assess with the direct, straight view afforded by a microscope. This has proven especially important in assessing completeness of dissection and searching for tumor remnants in hidden angles after the completion of tumor removal. While the minimally invasive nature of the EEA should not take priority over the radicality of dissection, it may help avoid the morbidity (scarring and blood loss) associated with a craniotomy, as well as the neurological sequelae associated with brain retraction [7].

However, EEA produces routinely large dural defects, frequently communicating directly with areas of high cerebrospinal fluid (CSF) flow leak, such as the third ventricle [7]. This was indeed the major limiting factor in the early days of EEAs, with CSF leak rates as high as 65% [7]. It is particularly of concern in cases of large suprasellar tumors such as the one reported here, as the large dural defect that may be required for adequate tumor removal may prove impossible to close in a watertight fashion. Several techniques have been suggested to solve this problem, including a variety of grafts, both autologous, heterologous or artificial, as well as vascularized pedicled flaps [8]. Other techniques have been described, such as suturing a fascial inlay patch using fascia obtained from the femur or abdomen, followed by a nasoseptal flap [9]. The use of a nasal septal pedicled flap combined with an autologous tissue graft has proven to be very useful for the repair of skull base defects during expanded endonasal endoscopic surgery, with postoperative CSF leaks as low as 7.7% [10]^ ^reported in some case series.

Other challenges in endoscopic skull base surgery are venous/arterial bleeding and the impression of a limited workspace, which limits the ability of the surgeon to work bimanually. However, the use of judicious hemostasis (with floseal and electro cautery) and adequate bony exposure can improve access significantly and reduce the sword fighting [7].

The significant risk of postoperative CSF leak associated with endoscopic resection of large midline tumors was mitigated in the reported case by placing a lumbar drain for CSF diversion and reconstructing the skullbase with a non-suturable dural allograft inlay, a nasoseptal flap, and dural sealant. While there haven’t been many reported cases of large suprasellar teratoma resection via the endoscopic endonasal approach, we present a case in which this approach was employed with favorable surgical and neurological outcome. 

## Conclusions

The endonasal endoscopic transphenoidal approach offers a minimally invasive approach that obviates the need for brain retraction and its associated temporary (and sometimes permanent) neurological injury. The incidence of postoperative CSF leak can also be significantly reduced with the use of a nasoseptal flap and dural allograft. It is a versatile approach, which may be successfully utilized for larger tumors including suprasellar germ cell tumors, as the operating surgeon’s comfort with the technique increases.
